# Highly Sensitive Label-Free Detection of Small Molecules with an Optofluidic Microbubble Resonator

**DOI:** 10.3390/mi9060274

**Published:** 2018-05-31

**Authors:** Zihao Li, Chenggang Zhu, Zhihe Guo, Bowen Wang, Xiang Wu, Yiyan Fei

**Affiliations:** Department of Optical Science and Engineering, Shanghai Engineering Research Center of Ultra-Precision Optical Manufacturing, Key Laboratory of Micro and Nano Photonic Structures (Ministry of Education), Fudan University, Shanghai 200433, China; 15210720012@fudan.edu.cn (Z.L.); 16110720026@fudan.edu.cn (C.Z.); 17110720004@fudan.edu.cn (Z.G.); 16210720012@fudan.edu.cn (B.W.)

**Keywords:** label-free sensor, optofluidic microbubble resonator, detection of small molecules

## Abstract

The detection of small molecules has increasingly attracted the attention of researchers because of its important physiological function. In this manuscript, we propose a novel optical sensor which uses an optofluidic microbubble resonator (OFMBR) for the highly sensitive detection of small molecules. This paper demonstrates the binding of the small molecule biotin to surface-immobilized streptavidin with a detection limit reduced to 0.41 pM. Furthermore, binding specificity of four additional small molecules to surface-immobilized streptavidin is shown. A label-free OFMBR-based optical sensor has great potential in small molecule detection and drug screening because of its high sensitivity, low detection limit, and minimal sample consumption.

## 1. Introduction

Methods to detect small molecular analytes (<1000 Da) have found several, important uses in the bio-medical, food, and environmental fields [1,2,3]. In biomedical analyses, several small molecules such as steroids, thyroid hormones, and peptides derived from disease-specific proteins have been utilized as diagnostic markers [4,5]. Additionally, the detection of small molecules analytes could serve a vital role in the identification of bacterial pathogens in infectious diseases [6,7].

To detect small molecules, several electrical, mechanical, and optical sensors have been developed, such as the nanomechanical resonator sensor, nanowire sensor, and surface plasmon resonance sensor. Among them, optical detection methods stand out because of their advantages, such as greater sensitivity, electrical passiveness, and robustness. To date, the most widely used optical detection technology uses labels, such as radio- or fluorescent-labeling, for detection. Although popular and useful, the labeling step requires additional time and cost, and even worse, a labeled approach can significantly alter the activities of small molecules and lead to inaccurate conclusions. Various label-free optical techniques, such as surface plasmon resonance (SPR) [8], resonant waveguide grating (RWG) [9], resonant mirror (RM) [10,11], and high-Q optical microcavities [12,13,14,15,16] have been developed, but their sensitivities diminish with the size of the molecule, making it extremely challenging to detect small molecules. To date, the detection limit for small molecules with label-free optical detection technologies are in the range of μM to nM [17,18].

An optofluidic microbubble resonator (OFMBR) that supports high-Q whispering gallery modes (WGMs) is a promising candidate for the development of sensors [19,20,21,22,23] because of its unique hollow-core structure, capabilities of integration with microfluidics, high sensitivity, and excellent optical confinement of the WGMs [24,25]. Light couples into the microbubble resonator through the fiber taper and the WGMs shift in response to the change of the refractive index in the medium surrounding the resonator, which leads to additional applications of OFMBR in label-free biosensing. However, the OFMBR is typically exposed in the air, which is susceptible to environmental changes, greatly diminishing its performance capabilities in small molecule detection.

In this paper, we propose a packaged OFMBR sensor by fixing the ends of a microbubble and fiber taper on glass substrate with glue, and sealing the OFMBR inside a glass box. By immobilization of streptavidin on the inner surface of microbubble and injection of various concentrations of small molecule biotin, we obtained real-time binding curves of biotin to immobilized streptavidin, which gave us a binding affinity of 6.7 × 10^14^ M^−1^. The detection limit of biotin to immobilized streptavidin on the packaged OFMBR sensor was determined to be 0.41 pM. In addition, we flew four small molecules over a streptavidin-immobilized OFMBR sensor; only magnolol bound to the surface-immobilized streptavidin, with binding affinity of 4.7 × 10^10^ M^−1^. The packaged OFMBR sensor was demonstrated to be able to detect small molecules with high sensitivity and low detection limit, which has significant application potential in small molecule detection and drug screening.

## 2. Materials and Method

### 2.1. Materials

The 3-glycidoxypropyltrimethoxysilan (GOPTS) was from Sinopharm Chemical Regent Company (Shanghai, China). Bovine serum albumin (BSA) and phosphate buffered saline (PBS) were from Sigma-Aldrich (St. Louis, MO, USA). Streptavidin (SA) was from Life Technologies (Shanghai, China). Biotin was from Aladdin Industrial Corporation (Shanghai, China). Magnolol, medetomidine HCl, cetrimonium bromide, and reboxetine mesylat were from Selleck (Houston, TX, USA). The low refractive index polymer MY133 was from MY Polymers (Ness Ziona, Israel). The UV glue was from Thorlabs (Shanghai, China).

### 2.2. The Packaged OFMBR Sensor for Biomolecular Interaction Detection

**Figure 1:**
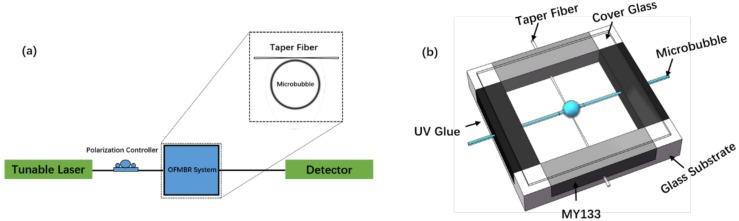
(**a**) Sketch of the detection system for the OFMBR sensor; (**b**) Structure of the packaged OFMBR sensor.

Figure 1a shows the sketch of the OFMBR detection system, which consists of a tunable, continuous diode laser, with a tuning range from 765 nm to 781 nm (TLB6700, New Focus, San Diego, CA, USA), a polarization controller (EPC300, Connet Fiber Optics, Shanghai, China), a packaged OFMBR sensor, a photon detector (DET10C, Thorlabs, Newton, NJ, USA), and an oscilloscope at a working sampling rate of 500,000 S/s (TDS3012, Tektronix, Portland, OR, USA). Figure 1b shows the structure of a packaged OFMBR sensor. The OFMBR was on a glass substrate, with wall thickness about 2–4 μm, lying perpendicular to a fiber taper; optimal coupling was achieved by adjusting the gap between them. To fix the position and wrap the fiber, low refractive index polymer MY133 was on both ends of the fiber taper. To fix the OFMBR on the substrate, UV glue (NOA68, Thorlabs, Newton, NJ, USA) covered both ends of the microbubble. In addition, a cover glass was placed on top of the four piles on the glass substrate corners to seal the packaged sensor. The packaged OFMBR sensor was solidified in the air by its exposure to a UV lamp at 365 nm for 5 min. Instead of putting MY133 around the coupling region of OFMBR with fiber taper and on the ends of the fiber taper as described before [25], we put polymer MY133 only on the ends of the fiber taper in such a way that excited WGMs were not affected; as a result, the as-packaged OFMBR sensor was more sensitive than that described before [25].

When biological molecules aggregated at the inner surface of the microbubble, they interacted with the evanescent part of the WGM field. Subsequently, a wavelength shift occurred as a result of the change of the radius or the refractive index of the microbubble. The resonance wavelength shift $\Delta_{\lambda }$ was proportional to the surface density of the small molecules $N_{\mathrm{AB}}\left(t\right)$, which is expressed as [26],

(1) $$\Delta_{\lambda }{\;=\;\alpha }_{\mathrm{ex}}\lambda S\frac{2\pi \sqrt{n_{2}^{2}-n_{3}^{2}}}{\varepsilon_{0}\lambda^{2}}\frac{n_{2}}{n_{3}^{2}}{\;\times \;N}_{\mathrm{AB}}\left(t\right){\;=\;\mu \;\times \;N}_{\mathrm{AB}}\left(t\right)$$

$n_{2},\;n_{3}$ are the refractive index values of the OFMBR’s wall (quartz) and the OFMBR’s core (air or liquid),${\;\alpha }_{\mathrm{ex}}$ is the excess polarizability of the biomolecules, S is the sensitivity of refractive index. By following the wavelength shift variations with time, binding kinetics of biomolecule to the OFMBR inner surface was revealed. 

### 2.3. Detection of Small Molecule Binding to Surface-Immobilized Streptavidin

**Figure 2:**
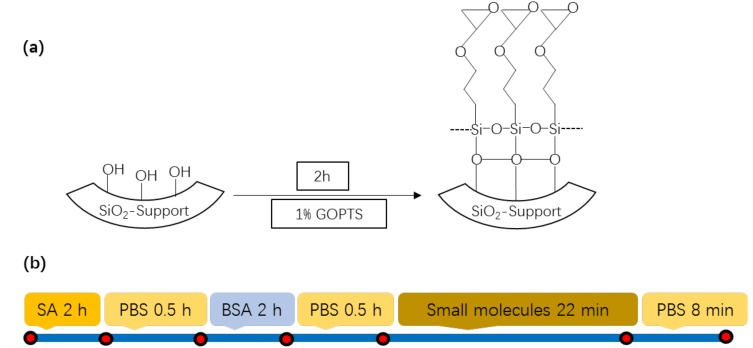
(**a**) Schematic illustration of surface functionalization of microbubble surface with epoxy groups; (**b**) Flow diagram of experimental processes for small biomolecule binding to surface-immobilized streptavidin.

Figure 2a schematically shows the functionalization of the inner surface of the microbubble with epoxy groups by using 3-glycidoxypropyltrimethoxysilan (GOPTS, Sinopharm Chemical Regent Company, Shanghai, China). The inner surface of OFMBR was first activated by 12% NaOH (Lingfeng Chemical Regent, Shanghai, China) for 1 h to obtain OH terminal groups. The microbubble was then filled with 1% GOPTS in 95% ethanol for 2 h, followed by washing with toluene, ethanol, and deionized water for 10 min each to remove residual unreacted GOPTS. The microbubble was finally dried in an oven at 120 °C for 2 h. The functionalized microbubble surface, having epoxy groups, formed covalent linkages with amine groups on proteins which were thus immobilized on the inner surface of microbubble with high efficiency.

Figure 2b shows experimental protocols for the detection of small molecule binding to surface-immobilized streptavidin. Protein streptavidin was first immobilized on the inner surface of the OFMBR by its incubation with streptavidin, at a concentration of 7.7 uM for 2 h, followed by 1 × PBS washing for 0.5 h. The inner surface was then blocked with 7.6 uM BSA at a flow rate of 2.5 μL/min for 2 h and followed by washing with 1 × PBS for 0.5 h.

Each binding curve of small molecule to surface-immobilized streptavidin included baseline, association phase, and dissociation phase. During baseline, 1 × PBS flew over surface-immobilized streptavidin at a flow rate of 2 μL/min for 6 min. During the association phase, small molecule solution flew over surface-immobilized streptavidin for 22 min at a flow rate of 2 μL/min. Dissociation phase included 1 × PBS flowing over the OFMBR at a flow rate of 2 μL/min for 8 min. Specific binding of small molecule to surface-immobilized streptavidin caused red-shift of the OFMBR WGMs, which was recorded from the beginning of the baseline to the end of the dissociation phase at a time resolution of 1 s. All binding curves were normalized against respective maximal resonance wavelength shift which does not affect reaction kinetic rate constants.

## 3. Results

### 3.1. Transmission Spectra and Q Factors of OFMBR Sensor

An OFMBR sensor consists of a microbubble coupled with a fiber taper. The OFMBR sensor exhibits high sensitivity due to the presence of a significant part of the WGM field close to the inner surface of the microbubble. In addition, the OFMBR sensor has excellent fluidic capability because of its intrinsic hollow structure. However, the exposed OFMBR sensor is susceptible to environmental changes and usually an exposed sensor lasts less than 12 h. We thus propose to package the OFMBR sensor within a sealed glass box (as described in Section 2.2) to reduce the impact of the environment.

**Figure 3:**
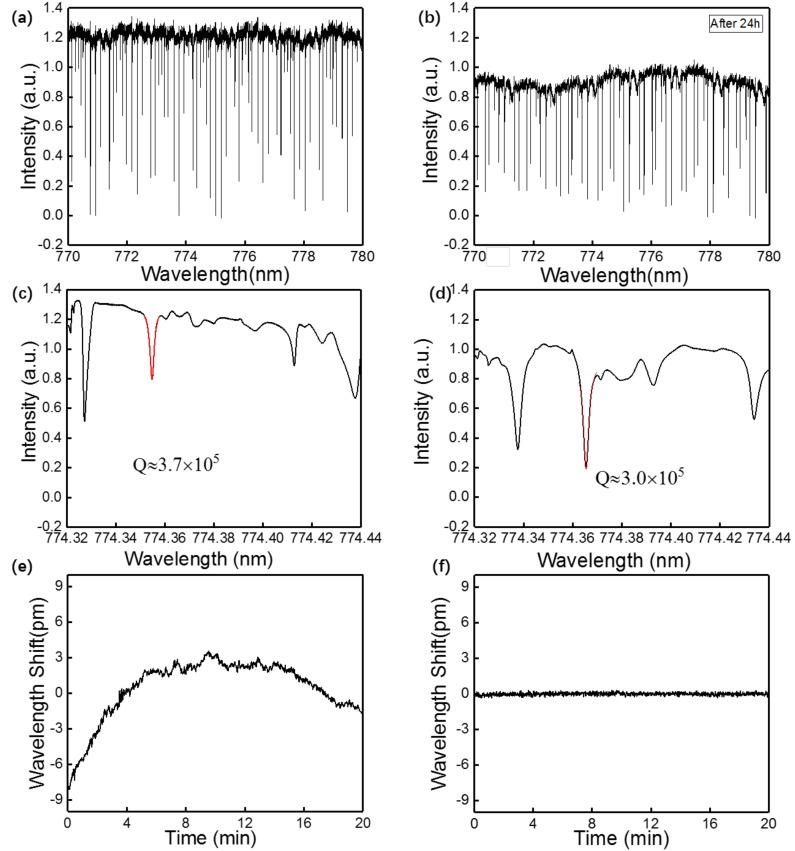
(**a**) Transmission spectra of an OFMBR before packaging in the wavelength range between 770 nm and 780 nm; (**b**) Transmission spectra of an OFMBR 24 h after packaging in the wavelength range between 770 nm and 780 nm; (**c**) Fine scan of the transmission spectra before packaging around 774.3 nm; (**d**) Fine scan of the transmission spectra after packaging around 774.3 nm; (**e**) Resonant wavelength shift of an OFMBR sensor varying with time before packaging; (**f**) Resonant wavelength shift of an OFMBR sensor varying with time after packaging.

Figure 3a,b show transmission spectra of an OFMBR sensor with air inside before packaging and 24 h after packaging within a wavelength range of 770–780 nm, respectively. After packaging, WGMs remained excited. The decreased intensity of the transmission light after packaging resulted from the loss of scattering and absorption due to the coating of MY133 around the ends of the fiber taper. Figure 3c,d show fine scan of the transmission spectra of OFMBR sensor before and after packaging around 774.3 nm. Both spectra show the same four WGMs, indicating that the excited WGMs were all reserved after the packaging process. The typical Q factors were obtained by Lorenz fits of the WGMs, indicated by the red curves in Figure 3c,d and the Q values before and after packaging were approximately 3.7 × 10^5^ and 3.0 × 10^5^, respectively. The Q values before and after packaging were on the same order of magnitude, proving good maintenance of Q values after the packaging. The slight decrease of Q values was attributable to changes of coupling coefficiency between the microbubble and the fiber taper, caused by a slight movement of taper fiber during the packaging process. Figure 3e,f show resonant wavelength shift varying with time of an OFMBR sensor before and after packaging. The standard deviation of resonant wavelength shifts decreased from 2.6 pm to 0.15 pm after packaging, indicating that sensor packaging greatly decreased resonant wavelength drift. In addition, performance of the as-packaged OFMBR sensor was stable for months. Results indicate that OFMBR sensor packaging could provide lower noise and longer lift time, without sacrificing its performance.

### 3.2. Binding Kinetics of Biotin to Surface-Immobilized Streptavidin

**Figure 4:**
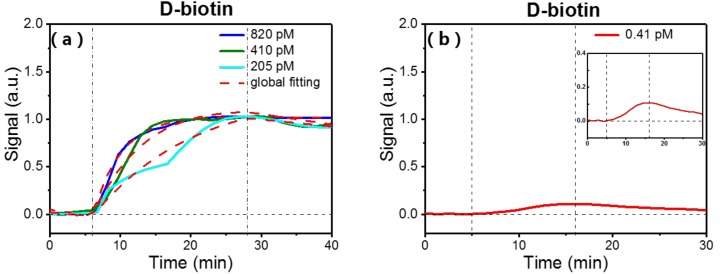
(**a**) Binding curves of surface-immobilized streptavidin with flowing biotin at respective concentrations of 205 pM, 410 pM, and 820 pM. Vertical lines indicate start of association and dissociation phases. Red dashed lines are global fitting results with the Langmuir reaction model; (**b**) Specific binding curve of surface-immobilized streptavidin with biotin at a concentration of 0.41 pM. Inset shows enlarged view of the binding curve.

Biotin is a small molecule with molecular weight of 224 Da, which binds strongly to streptavidin. We used biotin binding to surface-immobilized streptavidin as a model system to study the performance of a packaged OFMBR sensor on small molecule detection. We measured binding kinetics of biotin to surface-immobilized streptavidin at respective biotin concentrations of 205 pM, 410 pM, and 820 pM on three fresh OFMBR sensors; Figure 4a shows the three normalized real-time binding curves among them. Vertical lines indicate the start of the association and dissociation phases. Real-time binding curves contain information on kinetic rate constants, $k_{\mathrm{on}}$ (association rate) and $k_{\mathrm{off}}$ (dissociation rate). For the monophasic molecular interaction with stoichiometry 1:1, the affinity constant $k_{a}$ is the ratio of $k_{\mathrm{on}}$ to $k_{\mathrm{off}}$,

(2) $$A\;+\;B\overset{k_{\mathrm{on}}}{\to }\mathrm{AB}$$

(3) $$\mathrm{AB}\overset{k_{\mathrm{off}}}{\to }A\;+\;B$$

(4) $$k_{a}\;=\;\frac{k_{\mathrm{on}}}{k_{\mathrm{off}}}$$

To extract binding kinetic rate constants, we used a Langmuir reaction model. As shown in Equations (5), small molecules are assumed to bind to surface-immobilized streptavidin at a rate proportional to the association rate $k_{\mathrm{on}}$, small molecule concentration [A], and the unbonded binding sites $N_{{\left[\mathrm{AB}\right]}_{\max}}-N_{\left[\mathrm{AB}\right]}$ at time less than $t_{0}$. After the small molecule solution was replaced with a PBS buffer at time $t_{0}$, small molecule-protein complexes dissociated at a rate proportional to dissociation rate ${\;k}_{\mathrm{off}}$ and number of complexes per sensing area $N_{\left[\mathrm{AB}\right]}$.

(5) $$\{\begin{array}{c} \frac{{\mathrm{dN}}_{\left[\mathrm{AB}\right]}}{\mathrm{dt}}\;={\;k}_{\mathrm{on}}\left[A\right]\left(N_{{\left[\mathrm{AB}\right]}_{\max}}-N_{\left[\mathrm{AB}\right]}\right)-k_{\mathrm{off}}N_{\left[\mathrm{AB}\right]},t\;\leq {\;t}_{0},\\ \frac{{\mathrm{dN}}_{\left[\mathrm{AB}\right]}}{\mathrm{dt}}\;=\;-k_{\mathrm{off}}N_{\left[\mathrm{AB}\right]},t\;>{\;t}_{0} \end{array}$$

$N_{{\left[\mathrm{AB}\right]}_{\max}}$ is the maximal number of binding sites per sensing area. The number of small molecule complexes per unit sensing area $N_{\left[\mathrm{AB}\right]}$ is calculated to be,

(6) $$\{\begin{array}{c} N_{\mathrm{AB}}\left(t\right)\;=\;\frac{k_{\mathrm{on}}\left[A\right]N_{{\left[\mathrm{AB}\right]}_{\max\;}}}{k_{\mathrm{on}}\left[A\right]+k_{\mathrm{off}}}\left(1\;-e^{-(k_{\mathrm{on}}\left[A\right]+k_{\mathrm{off}})t}\right),t\;\leq \;t_{0},\\ N_{\mathrm{AB}}\left(t\right)\;=\;\frac{k_{\mathrm{on}}\left[A\right]N_{{\left[\mathrm{AB}\right]}_{\max\;}}}{k_{\mathrm{on}}\left[A\right]+k_{\mathrm{off}}}\left(1\;-e^{-(k_{\mathrm{on}}\left[A\right]+k_{\mathrm{off}})t_{0}}\right)e^{-k_{\mathrm{off}}\left(t-t_{0}\right)},t\;>\;t_{0}, \end{array}$$

We obtained reaction kinetic rate constants $k_{\mathrm{on}}$ and $k_{\mathrm{off}}$ by globally fitting three normalized binding curves in Figure 4a with Equations (6). As shown in Table 1, the binding affinity between small molecule biotin and surface-immobilized streptavidin was 6.7 × 10^14^ M^−1^, which is close to the affinity range (10^13^–10^14^ M^−1^) reported by others [27,28]. Figure 4b shows the binding curve of surface-immobilized streptavidin to flowing biotin at a concentration of 0.41 pM, which is significantly lower than the detection limit achieved on other label-free optical sensing systems [12].

### 3.3. Specificity of Small Molecules Binding to Surface-Immobilized Streptavidin

**Figure 5:**
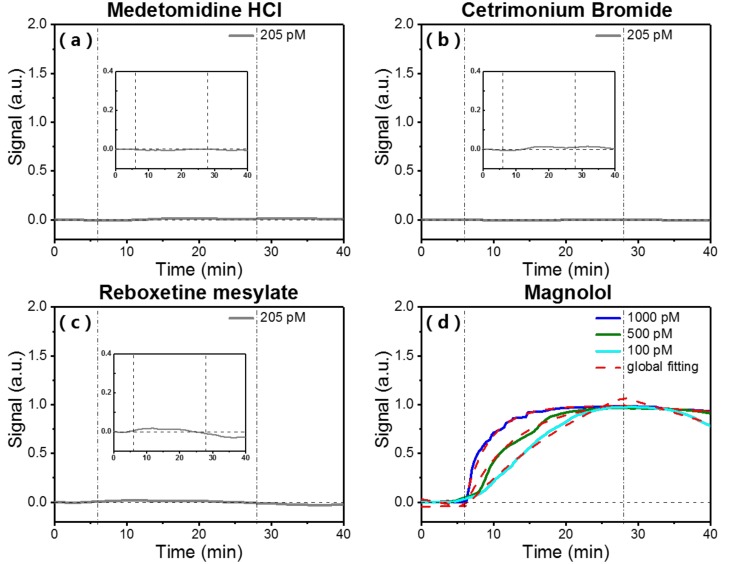
Real-time binding curves of small molecules (**a**) medetomidine HCl; (**b**) cetrimonium bromide; (**c**) reboxetine mesylate with surface-immobilized streptavidin on packaged OFMBR sensor at respective concentration of 205 pM (Insets show enlarged views of the binding curves); (**d**) Binding curves of surface-immobilized streptavidin with flowing magnolol at concentrations of 100 pM, 500 pM, and 1000 pM. Vertical lines are the starts of association and dissociation phases.

To study binding specificity of small molecules to surface-immobilized streptavidin on the packaged OFMBR sensor, we flew four small molecules at respective concentrations of 205 pM sequentially over the surface-immobilized streptavidin. Three small molecules, medetomidine HCl, cetrimonium bromided, and reboxetine mesylat were randomly selected from our compound library. Magnolol, a binding ligand of streptavidin screened from 3375 compounds [29], was also included. Figure 5 show that the three randomly selected small molecules did not bind to the surface-immobilized streptavidin and magnolol bound specifically to streptavidin, indicating that the as-packaged OFMBR sensor functionalized with streptavidin binds specifically with small molecules. From binding curves between streptavidin and magnolol at concentrations of 100 pM, 500 pM, and 1000 pM, the binding affinity between them was found to be 4.7 × 10^10^ M^−1^.

## 4. Discussion and Conclusions

The OFMBR sensor was demonstrated to be capable of detecting biomolecular interactions with high sensitivity, based on WGMs exited through a coupling of a microbubble resonator (OFMBR) and a fiber taper. Traditionally, a OFMBR sensor is exposed to the air and the sensor’s performance is susceptible to environmental changes, especially dirt in the air. In this way, the exposed OFMBR sensor usually lasts less than 12 h. In addition, it is not convenient to carry the exposed OFMBR sensor. To improve the stability of an OFMBR sensor, we fixed the ends of the sensor with glue on the glass substrate and put the packaged sensor inside a glass box. The as-packaged OFMBR sensor displayed good stability, lasting for months. A significant difference between the package protocols of this work and the package protocols described before [25] is that we did not wrap the coupling position between the microbubble and the fiber taper with MY133; accordingly, the high radial order modes were reserved to provide high sensitivity.

Detection limit of the packaged OFMBR sensor can be further optimized by enhancing sensor sensitivity, increasing surface density of immobilized protein ligand, and reducing noise level. Thin shells and high radial modes are beneficial for improving sensor sensitivity. Optimization of surface functionalization and surface immobilization protocols is expected to maximize protein surface density. Noise level can be reduced by increasing the stability of the OFMBR sensor, keeping it at a constant temperature, and using a self-referencing sensing scheme, such as mode-splitting method [14].

In this paper, we demonstrate a novel optical sensor for label-free small molecule detection with low detection limit. With surface-immobilized streptavidin on the inner surface of a packaged OFMBR sensor, binding of small molecule biotin to streptavidin can be detected with a reduced detection limit at 0.41 pM. The specificity of small molecule binding on OFMBR sensor was demonstrated with four additional small molecules. Such a sensitive optical sensor will find wide applications in medical diagnoses, treatment of diseases, and drug screening.

## Figures and Tables

**Table 1:** Kinetic constants of biotin and magnolol binding to immobilized streptavidin.

Small Molecule	k_on_ (min nM)^−1^	k_off_ (min)^−1^	k_a_ (M)^−1^
Biotin	0.30	4.5 × 10^−7^	6.7 × 10^14^
Magnolol	0.29	6.2 × 10^−3^	4.7 × 10^10^
